# Plant Sulfate Transporters in the Low Phytic Acid Network: Some Educated Guesses

**DOI:** 10.3390/plants8120616

**Published:** 2019-12-17

**Authors:** Gian Attilio Sacchi, Fabio Francesco Nocito

**Affiliations:** Dipartimento di Scienze Agrarie e Ambientali—Produzione, Territorio, Agroenergia, Università degli Studi di Milano, 20133 Milano, Italy; gianattilio.sacchi@unimi.it

**Keywords:** sulfate transporters, phytic acid, sulfur, phosphorous

## Abstract

A few new papers report that mutations in some genes belonging to the group 3 of plant sulfate transporter family result in low phytic acid phenotypes, drawing novel strategies and approaches for engineering the low-phytate trait in cereal grains. Here, we shortly review the current knowledge on phosphorus/sulfur interplay and sulfate transport regulation in plants, to critically discuss some hypotheses that could help in unveiling the physiological links between sulfate transport and phosphorus accumulation in seeds.

## 1. Background

Phytic acid (PA)—the major phosphorus (P) store in seeds—cannot be digested by humans and monogastric animals who lack the digestive enzyme phytase. For this reason, almost 90% of phytate consumed by humans is excreted, contributing to eutrophication of rivers, lakes, and oceans [1]. Furthermore, high levels of PA largely prevent the absorption of essential metals in the intestine, thus reducing further the nutritional value of the seeds [2,3].

In the last decades, several approaches have been proposed to solve the seed PA-related problems, including the engineering of crops for high phytase activity in seeds, or the selection of suitable *low phytic acid* (*lpa*) genotypes for crop breeding [4]. Today, numerous *lpa* genotypes have been identified and studied in several major crops, including maize, barley, wheat, rice, soybean, and common bean, reveling several mutations and alleles that could be potentially useful for breeding. However, a large part of the *lpa* phenotypes is caused by mutations in genes involved in PA biosynthesis or compartmentalization and often results in undesirable pleiotropic effects on yield-related traits and agronomic performances, since PA and inositol phosphates play pivotal roles in a plethora of developmental and signaling processes [4,5]. As a result, the use of these genetic resources to engineer seed PA content has proven to be challenging. Most recent advances in this research topic revealed that mutations in some members of the sulfate transporter gene family might result in *lpa* phenotypes. Unfortunately, little data are available to explain such effects fully or to develop new strategies for engineering seed PA content. Trying to fill this gap, here, we shortly review the current knowledge on plant sulfate transporters, trying to provide a glimpse into the complex and, in many respects, unexpected connections among the regulatory layers of sulfur (S) and P homeostasis in plants.

## 2. Sulfate Transporters: A Short Overview

S is an essential nutrient for plants. It is found in the amino acids cysteine and methionine, which are essential components of proteins and peptides, in vitamins and cofactors, and in a plethora of secondary compounds. S plays important and critical roles in a wide variety of cellular processes involved in plant development and response to environmental changes [6,7,8,9].

Sulfate (SO_4_^2−^) ions in the rhizosphere are the major source of S for plants. They are absorbed by roots and then allocated to different sinks by mean of specific sulfate transporters (SULTRs). The oxidized S atom in SO_4_^2−^ is then reduced and assimilated into cysteine, before entering other metabolic pathways, or directly used for sulfation reactions [9,10,11]. SULTRs are classified as H^+^/SO_4_^2−^ co-transporters, are integrated into membranes by 12 membrane-spanning domains, and contain a carboxyl-terminal region, named STAS (Sulfate Transporter/AntiSigma-factor), which is thought to be critical for both activity and stability of the transporters, as well as for their interaction with other proteins [9,12,13,14].

A multigene family encodes plant SULTRs. In the best-characterized species—*Arabidopsis thaliana* and, to a lesser extent, rice (*Oryza sativa* L.)—12 *SULTR* genes have been reported [14,15]. SULTRs can be divided into four functional groups or subfamilies, according to their amino acid sequences. The members of each group have specialized functions for SO_4_^2−^ uptake and distribution within the cells and among plant organs, as indicated by their different tissue and subcellular localization, and regulation pathways.

Group 1 of the family encodes high-affinity SULTRs. Two members of this group, SULTR1;1 and SULTR1;2, are mainly expressed in the outermost cell layers of the root (root hairs, epidermis, and cortex), where they largely contribute in determining the rate of SO_4_^2−^ uptake. Arabidopsis *sultr1;1sultr1;2* double-knockout lines are severely impaired in growth and unable to take up SO_4_^2−^ at low external concentrations [16,17,18,19]. Although these transporters seem to share the same function, they are differently regulated to fulfill the plant demand for S-containing compounds under different SO_4_^2−^ availabilities or soil conditions. In the currently accepted model, SULTR1;2 is thought to be the major component of the SO_4_^2−^ uptake system under normal S supply, whereas SULTR1;1 should play a most significant role under S deficiency or during other stresses [16,17,20,21,22].

Sulfate ions absorbed by root are translocated to shoot throughout the xylem and then distributed to different sink organs and tissues. It has been proposed that SULTR2;1, a low-affinity SULTR expressed in pericycle and xylem parenchyma, may play a pivotal role in controlling the amount of SO_4_^2−^ available to be loaded into the xylem, by acting as a scavenger reabsorbing the excess of the anion in the apoplastic space inside the root stele. Under S starvation, the increase in the transcript level of *SULTR2;1* could help in maintaining adequate fluxes of SO_4_^2−^ directed to the xylem [16,23]. It is important to note that a local expression of *SULTR2;1* has also been observed in the xylem parenchyma and phloem cells of the leaves, and that it is not possible to rule out that *SULTR2;1* transcript is also expressed below detection levels in the phloem companion cells of the root [16,24].

**Figure 1:**
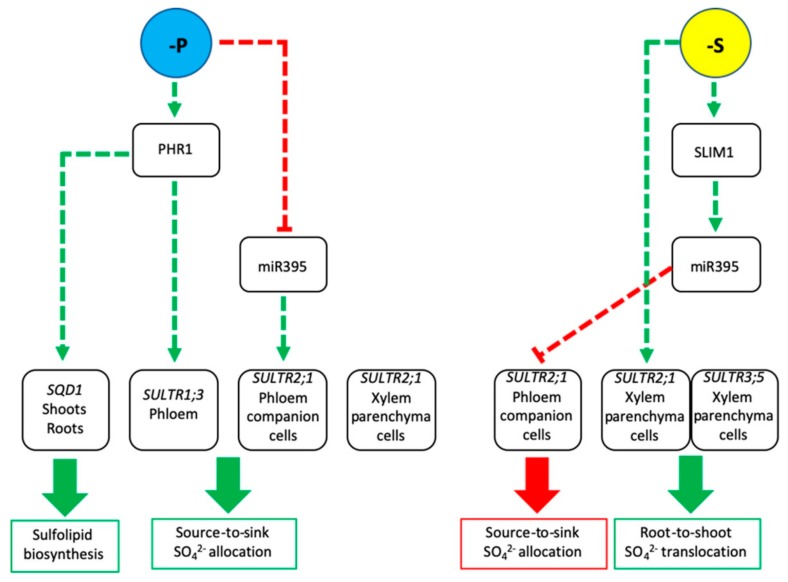
Main regulatory circuits controlling SO_4_^2−^ distribution in response to P or S status. Under S deficiency, the induction of *SULTR2;1*, in xylem parenchyma cells, and miR395, in phloem companion cells, enhances root-to-shoot SO_4_^2−^ translocation. In this condition, the co-expression of *SULTR3,5* could help the activity of SULTR2;1 in reabsorbing the excess of SO_4_^2−^ in the apoplastic space of the root. Under P deficiency, an extra regulatory circuit involving *PHR1* allows changes in SO_4_^2−^ to support sulfolipids biosynthesis.

An interesting regulatory circuit controls SO_4_^2−^ translocation and partitioning at the post-transcriptional level (Figure 1). The *SULTR2;1* mRNA is targeted and degraded by the miRNA-395 (miR395), which accumulates under S deficiency mainly in the companion cells of the phloem of both root and shoot [24]. The induction of miR395 is, in turn, activated by *SLIM1/EIL3* (*SULFUR LIMITATION 1/ETHYLENE-INSENSITIVE3-LIKE3*), a major regulator gene belonging to the EIL family transcription factors, which controls the expression of several S-responsive genes [25,26]. The mechanism by which miR395 controls *SULTR2;1* transcript level is not conventional, since the accumulation of both miR395 and *SULTR2;1* mRNA is induced under S starvation. However, the non-overlapping spatial expression domains of the two transcripts allows miR395 to restrict the expression of *SULTR2;1* to the xylem parenchyma cells of the root, thus inhibiting long-distance SO_4_^2−^ transport to sink tissues via the phloem and facilitating, at the same time, xylem SO_4_^2−^ translocation to the leaves [24,25].

Another low-affinity SULTR belonging to group 2, SULTR2;2, seems to be involved in controlling the source-to-sink distribution of SO_4_^2−^ inside the plant. Localization analyses indicate that SULTR2;2 may play a role in the transport of SO_4_^2−^ via root phloem, as well as in the distribution of the anion from leaf vasculature to the leaf palisade and mesophyll, which are thought to be the primary sites for SO_4_^2−^ assimilation [16]. Finally, long-distance transport of SO_4_^2−^ from source to sink organs could also involve SULTR1;3, a high-affinity SULTR of group 1, as indicated by the peculiar expression of this transporter in sieve elements and companion cells of the phloem [27].

Inside the cells, SO_4_^2−^ is further transported into the vacuole and chloroplast/plastid, where it is compartmentalized as S store or reduced and assimilated into cysteine for further metabolic processes, respectively. To date, tonoplast proteins mediating vacuolar SO_4_^2−^ influx have not been identified. On the other hand, SULTR4;1 and SULTR4;2 are known to be involved in downloading SO_4_^2−^ from the vacuoles under S limiting conditions [28].

Recently, all five members of group 3 have been indicated as redundantly involved in SO_4_^2−^ uptake across the chloroplast envelope membrane [29,30]. However, these observations do not appear to be conclusive, since several other functions could be postulated for these transporters on the base of observations that are crucial for our dissertation about the hypothetical links between *SULTRs* and *lpa* phenotypes. It is important to note that if, on the one hand, reasonable uncertainties about the capacity of both SULTR1s and SULTR2s to selectively move SO_4_^2−^ do not exist, on the other, no direct evidence has been provided about the actual SO_4_^2−^ transport activity of most of the SULTR3 subfamily members [14]. A few papers indeed indicate that both substrate preference and subcellular localization of some SULTR3s could be different than expected.

Kataoka et al. [31] reported that SULTR3;5 is expressed in the root vasculature of Arabidopsis—showing the same expression domain of the low-affinity SULTR2;1—and subcellular localizes on the plasma membrane. The heterologous expression of *SULTR3;5* in yeasts defective for SO_4_^2−^ uptake shows that this protein does not transport SO_4_^2−^ itself, whereas it enhances the SO_4_^2−^ uptake capacity of SULTR2;1 when co-expressed in the same yeast mutant. These results, along with the observation that the Arabidopsis *sultr3;5* mutant retains more SO_4_^2−^ in the root under S starvation, strongly suggest that SULTR3;5 may have the function to help SULTR2;1 in retrieval apoplastic SO_4_^2−^, contributing in this way to root-to-shoot SO_4_^2−^ translocation (Figure 1).

SULTR3;4 from rice and Arabidopsis have been recently indicated as SULTR-like phosphorus distribution transporters (SPDTs) playing essential roles in controlling the allocation of phosphate to grains and developing tissues, respectively [32,33]. Tissue-specific expression analyses show that SULTR3;4/SPDT of rice is expressed in the xylem region of both enlarged- and diffuse-vascular bundles of nodes [32]. The Arabidopsis ortholog gene shows a more complex expression pattern, since it is mainly expressed in the fascicular cambium between the xylem and phloem and in the interfascicular cambium of lower stem, as well as in the cambial zone of the leaf petiole, rosette basal region, hypocotyl, and in the parenchyma cells of both xylem and phloem surrounding the cambial zone [33]. Moreover, the SULTR3;4/SPDTs are localized at the plasma membrane, show proton-dependent transport activities for phosphate, do not transport SO_4_^2−^, and are up-regulated by phosphate deficiency but not under S starvation [32,33]. Mutations in *OsSULTR3;4/SPDT* alter the distribution of P in rice plants, decreasing both total P (−20%) and phytate (−30%) in the brown de-husked grains, without affecting yield, seed germination, and seedling vigor.

Another member of group 3, SULTR3;3, has been indicated as implicated in PA accumulation in barley and rice grains. Zhao et al. [34] recently reported that disruptions in rice *SULTR3;3* gene are the casual events of two interesting allelic mutations, previously described as *lpa-MH86-1* and *Os-lpa-Z9B-1*, since they produce grains with a reduced concentration of both PA and total P [35]. Tissue-specific expression analyses reveal that OsSULTR3;3 is expressed in the vascular bundles of shoots, leaves, flowers, and seeds, but not in the roots. This protein seems to be localized in the endoplasmic reticulum, when expressed in onion epidermal cells, and it does not show any transport activity for both SO_4_^2−^ and phosphate when heterologously expressed in yeast mutant strains defective for SO_4_^2−^ or phosphate uptake, or *Xenopus* oocytes. However—as underlined by Zhao et al. [34]—the lack of transport activity for SO_4_^2−^ or phosphate in heterologous systems does not necessarily mean that OsSULTR3;3 does not have a role in SO_4_^2−^ of phosphate transport, since its activity may depend on other proteins or post-translational modifications not present in non-plant hosts. Moreover, *OsSULTR3;3* mutations affect the concentrations of total P and phosphate of both root and shoot—which result higher in the mutants than in the wild type—but also reduce the concentrations of SO_4_^2−^ in the same organs. Finally, transcriptional analyses performed on developing grains reveal that *OsSULTR3;3* disruptions are associated with significant changes in the transcript level of genes involved in S and P homeostasis, suggesting a possible role of this gene in the cross-talk between the two nutrients [34]. Interestingly, a single base pair substitution in the last exon of an ortholog gene of *OsSULTR3;3* (designed as *HvST*) has also been identified as the causal event for the *low phytic acid* phenotype of the *lpa1-1* barley mutants [36].

Taken as a whole, these findings strongly indicate that expression domains and subcellular localizations, as well as substrate preferences of the SULTR3 subfamily members are variable, and may depend on plant species, development stage, or experimental approaches used to study their functions. Further efforts will be necessary to understand better whether this variability could play a role in the regulation of SO_4_^2−^ fluxes under different environmental conditions, also concerning the level of other essential mineral nutrients.

## 3. Sulfur and Phosphorous Interplay

Similar to S, P is also an essential macronutrient for plants. P is found as phosphate ester in the majority of the molecular constituents essential for plant cell functions, including nucleic acids, proteins, phospholipids, sugars, ATP, and NADPH. Important aspects related to P acquisition and homeostasis in plants have been recently reviewed elsewhere [37,38,39]. Here, we mainly focus our attention on S and P interplay by analyzing specific aspects related to SO_4_^2−^ transport and distribution inside the plants.

Although it is clear that S or P deficiencies have diverse phenotypic effects on plant growth, development, and productivity, intriguing interconnected responses to the internal levels of these two nutrients have been described at metabolic and transcriptional levels, suggesting the existence of coordination between S and P homeostasis. Rouached [40] pointed out that deficiency or surplus of only one of the two nutrients often results in changes in the expression levels of genes specifically involved in controlling the homeostasis of the other nutrient and underlined as comparable molecular mechanisms regulate both SO_4_^2−^ and phosphate transport in plants.

At the metabolic level, one of the most evident relationships between S and P is linked to membrane composition. It is known that cells can replace sulfolipids by phospholipids under S starvation, as well as they are able to replace phospholipids by sulfolipids and/or galactolipids under P starvation [41,42,43,44,45,46]. In Arabidopsis, the synthesis of sulfolipids is catalyzed by two enzymes, SQD1 and SQD2, whose expressions are increased by P starvation [42,43]. Although lipid shifts could be interpreted as adaptive mechanisms for plant survival under different nutrient availabilities, the physiological and biochemical consequences of phospholipids-sulfolipids substitutions on plant membrane functions are still unclear. Reprogramming membrane compositions under nutrient deficiency could have profound impacts on both S and P availability for plant metabolism. Moreover, recent studies have shown that the lipid environment and lipid-protein interactions may have crucial roles in modulating the functions as well as the conformational dynamics of membrane transporters [47].

Unfortunately, our basic knowledge about the interactions between P metabolism and SO_4_^2−^ transport is limited. A few papers show that P deficiency or perturbations in P metabolism may impact the SO_4_^2−^ allocation inside the plants. It has been reported that SO_4_^2−^ concentration increases in roots and decreases in shoots of Arabidopsis as a consequence of reduced phosphate availabilities in the growing medium [48]. Transcriptional analysis of the main *SULTR* genes implicated in long-distance SO_4_^2−^ transport reveals that, under P starvation, the transcript of *AtSULTR1;3* accumulates in both roots and shoots, whereas that of *AtSULTR2;1* weakly accumulates only in the roots. In the same conditions, *AtSQD1* transcript increases in both roots and shoots, indicating that adaptive modulations of SULTRs controlling the inter-organ distribution of SO_4_^2−^ are required for the replacement of phospholipid by sulfolipids induced by P starvation [48]. Most of these responses seem to be dependent on *PHR1* (*PHOSPHATE RESPONSE1*), a gene encoding a protein belonging to the MYB-CC family transcription factors involved in the activation of several phosphate starvation-induced genes (PSI) [38,49]. PHR1 binds to an imperfect palindromic motif, named P1BS, which is prevalent in the promoter of the PSI genes [49,50]. Interestingly *cis*-regulatory motifs for PHR1-dependent gene activation are also present in the promoters of both *AtSULTR1;3* and *AtSQD1* genes, whose expressions are coherently reduced in the Arabidopsis *phr1* mutant grown under P starvation [48,51,52]. Interestingly, other evidence indicates *PHR1* as the convergent point for the cross-talk between P and other essential nutrients, such as zinc and iron [53,54]. *AtSULTR2;1*, is up-regulated by P starvation in a PHR1-independent manner, since *AtSULTR2;1* transcript further accumulates in *phr1* P deficient plants [48]. In this context, the observation that the expression of miR395—the microRNA that mainly controls the spatial expression of *SULTR2;1* in vascular tissues—is suppressed under P deficiency, allows us to speculate about the existence of an extra regulatory circuit which controls the inter-organ distribution of SO_4_^2−^ under P starvation [55]. In this circuit (Figure 1): (i) the suppression of miR395 should allow SULTR2;1 to control root-to-shoot SO_4_^2−^ translocation via the xylem route, as well as the source-to-sink SO_4_^2−^ re-allocation via the phloem; (ii) PHR1 activates the expression of SULTR1;3 increasing further the capacity of the plants to move SO_4_^2−^ from source to sink tissues. Unfortunately, no other evidence is available to support this extra regulatory circuit further and to fully appreciate its possible physiological impact on S metabolism in P deficient plants. Finally, the observation that Arabidopsis lines engineered for low PA content show alterations in SO_4_^2−^ distribution and changes in expression of some *SULTRs* suggests the existence of another level of complexity in the cross-talk between S and P, which directly involves PA [56].

## 4. SULTRs as Novel Elements in the *lpa* Network

As mentioned above, genetic lesions in some genes putatively involved in sulfate transport result in *lpa* phenotypes in rice and barley [32,34,36]. Interestingly, all the mutations described thus far affect putative SULTR genes belonging to the SULTR3 subfamily, which includes elements whose functions are still objects of debate. For a detailed description of the *SULTR3/lpa* alleles, readers are referred to Cominelli et al. [57].

Differently from other SULTRs, whose capability to transport SO_4_^2−^ has mostly been proven using yeast mutants as heterologous expression systems, the function of the SULTR3s as SO_4_^2−^ transporters has only been hypothesized on the base of their sequence homologies with other SULTRs. Moreover, species-specific differences could explain the variability observed for the subcellular membrane localization of SULTR3 subfamily members.

Mineral nutrients required for plant growth are absorbed by the roots from the soil solution and then released to the xylem to be translocated to different tissues together with the transpiration flow. However, transpiration cannot be considered as the sole driving force for the root-to-shoot movement of nutrients, since developing organs such as new leaves and seeds are not photosynthetically active. Recently, nodes of gramineous plants have been identified as the main actors controlling nutrient delivery to developing tissues in a transpiration-independent way [58,59]. Several rice transporters involved in the intervascular transfer of nutrients from enlarged vascular bundles to diffuse vascular bundles of nodes seem to be essential to ensure this process [59]. Among these, *OsSULTR3;4/SPDT* has been indicated as pivotal in controlling phosphate delivery to developing tissues since it shows a proton-dependent transport activity for phosphate (but not for SO_4_^2−^), and it is highly expressed in the node 1 of rice at the reproductive stage [32]. Moreover, *OsSULTR3;4/SPDT* knockout mutants reduce P allocation to new leaves and grains, raveling the essential role of this transporter in switching phosphate toward developing leaves and grains.

The recent finding that the Arabidopsis *SULTR3;4* ortholog gene also controls xylem-to-phloem phosphate transfer, strongly suggests that sequence homology of SULTR3;4 with other SULTRs does not necessarily indicate that they share the same function [33]. All these observations not only show SULTR3;4s as phosphate transporters rather than as SO_4_^2−^ transporters, but may also explain the role of these proteins in the *lpa* network.

If, on the one hand, the recent description of SULTR3;4/SPDTs as phosphate transporters seems to leave no room for doubt, on the other, assessment of the role of SULTR3;3s on P allocation still appears challenging. Rice SULTR3;3 is mainly expressed in vascular tissues and does not show any transport activity for SO_4_^2−^ and phosphate [34]. Further studies are thus needed to uncover its function and subcellular localization. However, the lack of specific transport activity for SO_4_^2−^ has also been indicated for the Arabidopsis SULTR3;5, which has been described as an essential component of the SO_4_^2−^ transport system that facilitates the root-to-shoot SO_4_^2−^ translocation in the vasculature [31]. Although the mechanisms controlling SO_4_^2−^ allocation in rice are still known, it is possible to speculate that also *OsSULT3;3* could have a role in SO_4_^2−^ partitioning among organs, by helping the activity of some other vascular transporter, or in SO_4_^2−^ transport into the chloroplast, as recently suggested for its ortholog in Arabidopsis [30]. Rice *sultr3;3* mutants show significant alterations in S and P homeostasis, as indicated by the reduced concentration of SO_4_^2−^ in both shoots and roots, as well as by the accumulation of transcripts of several S- and P-responsive genes in developing grains. Disruption in *OsSULTR3;3* also affects the concentrations of various grain metabolites not directly involved in PA biosynthesis. In particular, the reduced level of cysteine, along with the accumulation of its precursor serine, seems to indicate an insufficient supply of S during seed differentiation. Interestingly, reduced levels of cysteine have also been observed in the chloroplasts isolated from different Arabidopsis *sultr3* mutants [30]. Thus, the alterations in S homeostasis could be interpreted as the primary physiological event that reduces the accumulation of P in the grains of *sultr3;3* mutants. Finally, since total P and phosphate concentrations in root and shoot are higher in mutants than in the wild type, we may further speculate about the existence of mechanisms that somehow limit the systemic mobility of P in the plant. The analysis of the membrane lipid composition could provide in the next future a possible explanation for this phenomenon since substitution of sulfolipids by phospholipids caused by an insufficient S supply could increase the amount of P immobilized within cell membranes. 

## 5. Conclusions and Perspectives

The implication of SULTRs in seed P accumulation not only provides novel opportunities to design routes for the breading of new *lpa* varieties in important cereal crops but also reveals the existence of a complex network of interactions between S and P homeostasis. The recent finding showing the involvement of *SULTR3;4/SPTDs* in delivering phosphate, and not sulfate, to developing tissues opens new questions about the nature of the other members of the SULTR3 subfamily [32,33]. Further investigations aimed at determining their substrate preference between sulfate and phosphate are then essential to unveil the actual role of these transporters in the control of nutrient homeostasis. In this context, the recent study of Cao et al. [29], suggesting that all the Arabidopsis *SULTR3* homologs may redundantly mediate sulfate import into the chloroplast, needs to be carefully reconsidered since chloroplasts isolated from the *sultr3* quintuple mutant retain about 50% of the sulfate uptake capacity of the wild type. Redundancy versus diversity will be the novel challenge to face.
